# Bicarbonate Transport in Cystic Fibrosis and Pancreatitis

**DOI:** 10.3390/cells11010054

**Published:** 2021-12-24

**Authors:** Dora Angyal, Marcel J. C. Bijvelds, Marco J. Bruno, Maikel P. Peppelenbosch, Hugo R. de Jonge

**Affiliations:** Department of Gastroenterology and Hepatology, Erasmus MC University Medical Center Rotterdam, P.O. Box 2040, 3000 CA Rotterdam, The Netherlands; d.angyal@erasmusmc.nl (D.A.); m.bijvelds@erasmusmc.nl (M.J.C.B.); m.bruno@erasmusmc.nl (M.J.B.); m.peppelenbosch@erasmusmc.nl (M.P.P.)

**Keywords:** CFTR, cystic fibrosis, pancreatitis, bicarbonate

## Abstract

CFTR, the cystic fibrosis (CF) gene-encoded epithelial anion channel, has a prominent role in driving chloride, bicarbonate and fluid secretion in the ductal cells of the exocrine pancreas. Whereas severe mutations in *CFTR* cause fibrosis of the pancreas in utero, CFTR mutants with residual function, or CFTR variants with a normal chloride but defective bicarbonate permeability (CFTR^BD^), are associated with an enhanced risk of pancreatitis. Recent studies indicate that CFTR function is not only compromised in genetic but also in selected patients with an acquired form of pancreatitis induced by alcohol, bile salts or smoking. In this review, we summarize recent insights into the mechanism and regulation of CFTR-mediated and modulated bicarbonate secretion in the pancreatic duct, including the role of the osmotic stress/chloride sensor WNK1 and the scaffolding protein IRBIT, and current knowledge about the role of CFTR in genetic and acquired forms of pancreatitis. Furthermore, we discuss the perspectives for CFTR modulator therapy in the treatment of exocrine pancreatic insufficiency and pancreatitis and introduce pancreatic organoids as a promising model system to study CFTR function in the human pancreas, its role in the pathology of pancreatitis and its sensitivity to CFTR modulators on a personalized basis.

## 1. Introduction

Cystic fibrosis (CF) is a potentially fatal multi-organ disease caused by mutation of the cystic fibrosis transmembrane conductance regulator (*CFTR*) gene. It is estimated that CF currently affects >100,000 people worldwide, and that by 2025, the number of patients will increase by 50% [1,2]. *CFTR* encodes a phosphorylation-regulated, adenosine triphosphate (ATP)-gated, epithelial anion channel that mediates chloride (Cl^−^) and bicarbonate (HCO_3_^−^) transport across epithelia, principally located in the respiratory, gastrointestinal and male reproductive tracts. The term cystic fibrosis refers to the CF-typical fibrotic lesions in the pancreas, first described in the 1930s [3]. In approximately 85% of CF patients, fibrosis of the pancreas starts in utero, progressing to a complete loss of exocrine pancreas function soon after birth, i.e., exocrine pancreatic insufficiency [4]. The severely reduced release of HCO_3_^−^ and digestive enzymes into the upper intestinal tract impairs the neutralization of gastric acid and causes malabsorption of nutrients.

Pancreatitis is a complex inflammatory disease of the acinar and ductal epithelia, which probably results from premature activation of digestive enzymes [5]. Currently, there is no specific therapy available, and treatment relies on supportive care. Pancreatitis is one of the three most common causes of gastrointestinal disease-related hospitalizations and is associated with high morbidity and mortality [6]. Acute pancreatitis (AP), recurrent AP (RAP) and chronic pancreatitis (CP) are thought to represent a disease continuum [7]. As opposed to the monogenic disease CF, in pancreatitis, multiple (epi-)genetic, metabolic and environmental factors combine and lead to a complex pathology [8,9]. A substantial body of evidence indicates that the loss of ductal CFTR-dependent fluid and HCO_3_^−^ secretion may precipitate the development of pancreatitis [10]. This is perhaps most poignantly illustrated by the fact that pancreas-sufficient CF patients, i.e., those who have retained some level of exocrine pancreatic function, are at increased risk of developing (R-)AP and CP [11]. Furthermore, it has been shown that carriers of a CF allele are at increased risk of developing pancreatitis [12].

CFTR-mediated HCO_3_^−^ secretion not only drives osmotic fluid secretion in ductal structures but is also required for controlling the pH at the epithelial surface, and the proper expansion of secreted mucins. Thus, in CF, a lowering of the luminal pH is thought to result in the accumulation of a hyper-viscid mucus that blocks the ductal structures and promotes microbial colonization and inflammation. In addition, in the pancreas, high levels of HCO_3_^−^ are thought to maintain secreted digestive enzymes in an inactive state while still located in the ductal tree [13].

In view of the apparent relevance of HCO_3_^−^ in the pathophysiology of CF and CFTR-related disorders (CFTR-RD) such as pancreatitis, it is perhaps surprising that most research into the function of CFTR has focused on its role as a Cl^−^ channel [14]. Perhaps due to the fact that it is readily formed from and converted to CO_2_ and H_2_O, accurate assessment of HCO_3_^−^ concentrations and transport is challenging, which may explain why CF biomarkers and therapy testing are Cl^−^ biased [15]. This is true for clinical nasal potential difference (NPD) and sweat Cl^−^ tests which are Cl^−^ based, as well as CFTR modulator testing which is based on iodide quenching or membrane potential measurements, while intestinal current measurements (ICMs) and forskolin-induced swelling (FIS) assays measure combined Cl^−^ and HCO_3_^−^ transport.

This review summarizes our current knowledge of the role of CFTR in pancreatic ductal fluid and HCO_3_^−^ transport, and the role of CFTR dysfunction in pancreatitis. As is outlined in the next section, CFTR is a critical element of the ion transport machinery in ductal epithelial cells, which, remarkably, is able to accumulate HCO_3_^−^ in human pancreatic juice to levels of up to 140 mmol/L. Several seminal studies published in the last decade point towards a prominent role of impaired CFTR-mediated HCO_3_^−^ transport in the pathophysiology of pancreatitis [16,17]. This offers the encouraging prospect that CFTR modulators, originally developed for treatment of CF, may also be of potential benefit for other pancreas-related diseases.

## 2. Bicarbonate Transport in the Exocrine Pancreas

### 2.1. CFTR Is Indispensable for the Accumulation of Bicarbonate in Pancreatic Juice

In the exocrine pancreas, acinar cells secrete various digestive enzymes in a small volume of isotonic, NaCl- and H^+^-rich fluid, after which the ductal epithelium serves to exchange the secreted Cl^−^ for HCO_3_^−^, to produce an alkaline fluid (pH 8.0–8.5) [13,18,19]. It is estimated that the human exocrine pancreas secretes up to 1 L of pancreatic juice per day [18,20,21]. Apart from delivering digestive enzymes to the proximal small intestine, making it essential for digestion of (macro-)nutrients, pancreatic juice serves to supply base equivalents for neutralization of the gastric acid entering the small intestine from the stomach. In humans, the HCO_3_^−^ concentration in pancreatic juice can reach levels of 140 mmol/L [22,23]. Similarly high levels of HCO_3_^−^ were observed in pancreatic juice of guinea pigs, whereas, in contrast, in mice and rats, HCO_3_^−^ levels did not exceed 70 mmol/L [24,25,26,27,28].

In humans, the production of pancreatic juice is strongly dependent on postprandial release of the hormone secretin, whereas production is low during fasting. Secretin, through activation of Gα,s-coupled receptors, triggers cyclic AMP (cAMP) production and a consequent protein kinase-mediated phosphorylation and activation of CFTR. In humans, CFTR-mediated, cAMP-dependent ductal fluid secretion is strongly potentiated by parasympathetic, vagal stimulation. Most plausibly, the activation of muscarinic (Gα,q) receptors by acetylcholine triggers K^+^ efflux through Ca^2+^-dependent K^+^ channels, which sustains the negative electrical potential across the luminal plasma membrane driving Cl^−^ and HCO_3_^−^ efflux through CFTR. In addition to cholinergic neurons, the parasympathetic system comprises neurons that produce vasoactive intestinal polypeptide (VIP), which, as with secretin, activates Gα,s-coupled receptors.

### 2.2. Molecular Mechanisms of CFTR-Dependent Bicarbonate Transport

**Figure 1:**
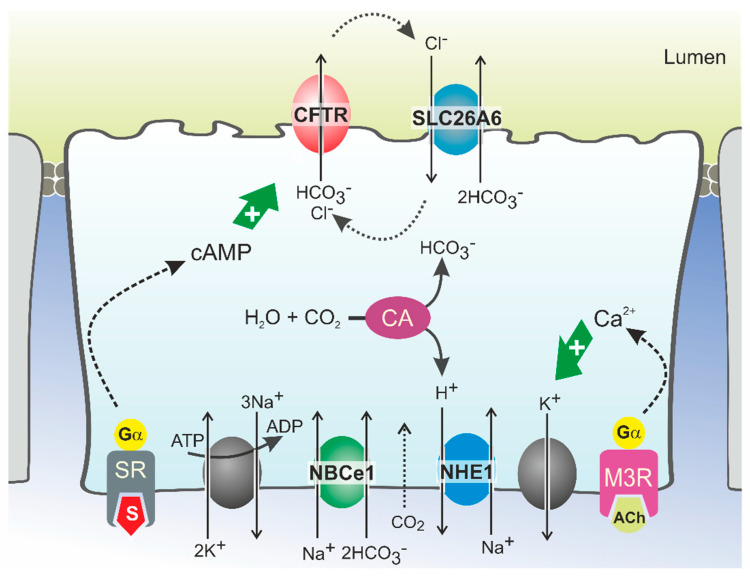
Model of HCO_3_^−^ secretion in pancreatic ducts. HCO_3_^−^ secretion is controlled by neuro-endocrine input, chiefly by secretin (S) and acetylcholine (ACh), which increase cellular cAMP and Ca^2+^ levels, respectively, via stimulation of G protein-coupled receptors. This triggers Na^+^-coupled HCO_3_^−^ uptake across the basolateral plasma membrane through NBCe1 and luminal HCO_3_^−^ efflux through CFTR and SLC26A6. HCO_3_^−^ may also be accumulated intracellularly via the coordinated activity of carbonic anhydrases (CA) and a proton extrusion mechanism, NHE1. Opening of K^+^ channels sustains the negative membrane potential which drives (CFTR-mediated) anion efflux. Cl^−^ ions absorbed via SLC26A6 may be recycled to the ductal lumen via CFTR. Ultimately, ductal ion and fluid secretion is driven by ion and electrical gradients that are maintained by Na^+^,K^+^-ATP-ase, located in the basolateral membrane. SR: secretin receptor; M3R: muscarinic acetylcholine receptor 3.

HCO_3_^−^ secretion involves the coordinated activity of ion transporters located at the basolateral and luminal membranes of pancreatic ductal cells (PDCs) (Figure 1). According to current models, which are discussed in detail elsewhere, uptake of HCO_3_^−^ from the interstitial space is principally mediated by a Na^+^/HCO_3_^−^ cotransporter (NBCe1, *SLC4A4*) located in the basolateral plasma membrane [13,29]. This transport is driven by the steep electrochemical Na^+^ gradient across the plasma membrane (generated by Na^+^,K^+^-ATPase activity) and allows for intracellular accumulation of HCO_3_^−^ up to levels well above electrochemical equilibrium. In addition, HCO_3_^−^ may be produced intracellularly from carbonic anhydrase-catalyzed hydration of CO_2_, which enters the cells through diffusion (Figure 1). While the protons produced in this reaction are extruded across the basolateral plasma membrane via a Na^+^/H^+^ exchange mechanism, HCO_3_^−^ is secreted across the apical membrane into the ductal lumen via the coordinated activity of the CFTR channel and the Cl^−^/HCO_3_^−^ exchangers SLC26A6 and, more tentatively, SLC26A3 [13,30,31,32]. According to this model, which may also be relevant for other HCO_3_^−^-secreting epithelia such as the intestinal and biliary epithelia, CFTR serves to facilitate Cl^−^/HCO_3_^−^ exchanger-mediated HCO_3_^−^ secretion by extruding the Cl^−^ ions absorbed through the exchangers [22,30,33,34]. As for CFTR, the activity of Cl^−^/HCO_3_^−^ exchangers is stimulated by cAMP and Ca^2+^ agonists, which implies that their activity is also controlled through neuro-endocrine stimulation of Gα,s- and Gα,q-coupled receptors [35]. CFTR may not only couple functionally to SLC26A6, but also physically, through binding of its regulatory (R) domain to the sulfate transporter anti-sigma (STAS) domain of SLC26A6 [36,37]. This interaction was shown to promote Cl^−^/HCO_3_^−^ exchange, even in the absence of CFTR activity [22,36].

Although there is ample evidence for the role of SLC26-type Cl^−^/HCO_3_^−^ exchangers in ductal HCO_3_^−^ secretion, mathematical modeling of the ion motive forces in the ductal epithelium predicted that Cl^−^/HCO_3_^−^ exchange mechanisms only significantly contribute to cellular HCO_3_^−^ extrusion up to luminal HCO_3_^−^ levels of circa 70 mmol/L [29,38]. Consequently, SLC26-type HCO_3_^−^ transport mechanisms may serve to secrete HCO_3_^−^ in the proximal segments of the ductal tree, but to reach the final, high HCO_3_^−^ levels observed in human pancreatic juice, an alternative transport mechanism must prevail in the distal part. In view of these considerations, it seems plausible that CFTR has a direct role in ductal HCO_3_^−^ secretion. The CFTR channel itself was shown to directly mediate cellular HCO_3_^−^ transport in studies on both cell models and epithelial tissues, including pancreatic ducts [39,40,41,42]. It has, however, also been noted that the permeability of the CFTR channel pore for HCO_3_^−^ is 4–5 times lower than for Cl^−^ [39,40,43,44]. This implies that CFTR can only selectively secrete HCO_3_^−^ when very low intracellular Cl^−^ and comparatively high HCO_3_^−^ concentrations are attained. This situation may occur in the distal part of the ductal tree where, upon hormonal stimulation of pancreatic secretion, Cl^−^ levels in PDCs decrease to values as low as 5 mmol/L [29]. In these settings, assuming that, concurrently, intracellular HCO_3_^−^ is actively accumulated through NBCe1-mediated uptake, it is conceivable that CFTR may function primarily, if not exclusively, as a HCO_3_^−^ transporter. 

To account for the high HCO_3_^−^ and low Cl^−^ levels found in pancreatic juice, it has been further proposed that the Cl^−^ over HCO_3_^−^ conductance ratio of CFTR is not static but can be lowered to allow selective HCO_3_^−^ secretion [45,46,47,48]. This dynamic regulation of CFTR anion conductance is thought to be mediated by members of the WNK (with no lysine) type of protein kinases [13,18,49,50]. WNK kinases (the family consists of four members) regulate an array of ion transporters through activation of downstream protein kinases OSR1 (oxidative stress-responsive kinase 1) and the highly homologous SPAK (STE20/SPS1-related proline/alanine-rich kinase) [51]. The WNK kinases are thought to function as osmotic stress sensors and are activated by a lowering of intracellular Cl^−^ levels. Notably, WNK1 and WNK4 control renal electrolyte transport, and mutations in either cause hypertension as a result of an unchecked tubular Na^+^ re-absorption. In most cases, WNK kinases regulate the activity of ion transport mechanisms by controlling their localization in the plasma membrane. 

**Figure 2:**
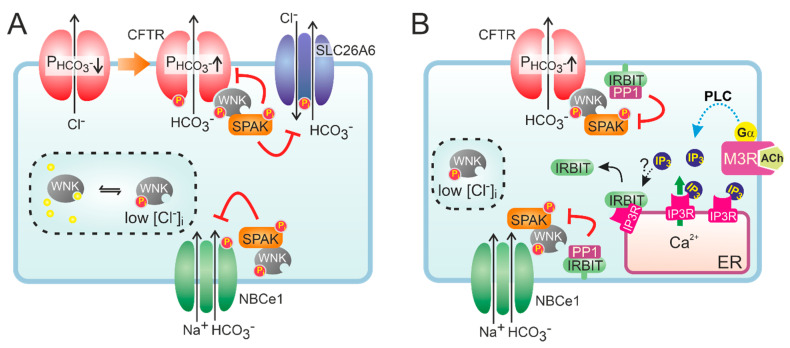
Hypothetical model of the role of WNK and IRBIT in ductal HCO_3_^−^ secretion. (**A**) Activation of CFTR decreases intracellular Cl^−^, which relieves the inhibition imposed by Cl^−^ on WNK and stimulates auto-phosphorylation of the protein kinase. Activated WNK binds to CFTR and increases the HCO_3_^−^ permeability (P) of the channel pore, independent of its protein kinase activity. However, WNK also recruits SPAK, which leads to a phosphorylation-mediated decrease in the cell surface expression of HCO_3_^−^ transporters, including CFTR. (**B**) IRBIT is bound to IP_3_ receptors (IP3R) on cellular Ca^2+^ stores, e.g., the endoplasmic reticulum (ER). Conceivably, IRBIT is displaced from IP3R upon a phospholipase C (PLC)-mediated increase in IP_3_ production, after which it translocates to the plasma membrane. Through its PDZ domain, IRBIT anchors to the actin cytoskeleton (not shown) and recruits a protein phosphatase (PP1) to the WNK/SPAK complex. PP1 activity counteracts SPAK-dependent phosphorylation of HCO_3_^−^ transporters, thus stabilizing their expression at the cell surface. IRBIT does not affect the WNK-dependent but rather the SPAK-independent increase in CFTR HCO_3_^−^ permeability.

At low extracellular Cl^−^ concentrations, WNK1-mediated OSR1 and SPAK activation was shown to markedly increase the HCO_3_^−^ permeability of CFTR in CFTR-transfected HEK293 cells and in guinea pig PDCs [52]. Interestingly, WNK1 activation depended on CFTR function as the change in HCO_3_^−^ permeability was not observed in cells expressing non-functional, mutant (F508del) CFTR, suggesting CFTR is required for lowering of the intracellular Cl^−^ concentration. More recently, it was proposed that WNK1 increases the HCO_3_^−^ permeability of CFTR by a direct interaction that does not require SPAK activation (Figure 2) [53]. WNK kinases are also thought to reduce the surface expression of Cl^−^/HCO_3_^−^ exchangers of the SLC26A family [54]. At high luminal HCO_3_^−^ levels such as those encountered in the distal ductal tree, this silencing of Cl^−^/HCO_3_^−^ exchange activity may prevent re-uptake of HCO_3_^−^ and, consequently, promote its net secretion. Paradoxically, both WNK1 and WNK4 were also shown to reduce CFTR protein levels at the cell surface and suppress CFTR-mediated anion efflux [55]. Moreover, WNK kinases may also reduce NBCe1 activity, suggesting that their activation opposes CFTR-mediated HCO_3_^−^ secretion [56]. However, the inhibition imposed by the WNK/SPAK pathway on ductal fluid and HCO_3_^−^ secretion is counteracted by the action of the scaffolding protein inositol-1,4,5-trisphosphate (IP_3_) receptor-binding protein released with IP_3_ (IRBIT) [56]. Through its PDZ domain, IRBIT co-localizes at the apical and basolateral plasma membrane with CFTR and NBCe1, respectively, and prevents WNK1/SPAK-mediated endocytosis by recruiting a protein phosphatase (PP1) that counters SPAK-mediated phosphorylation of CFTR and NBCe1 [56,57,58]. In addition to its effect on surface expression, IRBIT may also increase the CFTR open channel probability [59]. Recruitment of IRBIT to these molecular complexes appears to be stimulated through cAMP and Ca^2+^ signaling, and IRBIT is required for the synergism observed between these signaling pathways [60,61].

## 3. Bicarbonate Transport in Subjects with *CFTR* Mutations

The past decade saw great advances in understanding the genetics of pancreatitis, including the role of *CFTR* mutations. Genes in which mutation confers an increased risk of developing (chronic) pancreatitis are grouped in three categories: (1) the trypsin-dependent pathway including *PRSS1*, *PRSS2*, *SPINK1*, *CTRC* and *CTRB1* [62,63,64]; (2) the misfolding-dependent pathway including *CPA1* [65] and *CEL* [66]; and (3) the ductal pathway which, aside from *CFTR*, includes *CLDN2* and *CASR* [5]. *CFTR* mutations are associated with development and earlier age of onset of RAP [67,68,69] and CP [70,71,72,73]. In a large pediatric cohort, 34% of RAP and 23% of CP patients carried *CFTR* mutations [74]. In a group of RAP patients with a mean age of 23 years, 15% carried *CFTR* mutations [69], and in a cohort of idiopathic CP patients, this number was nearly 20% [72]. Up to 7% of all CP patients carry *CFTR* mutations, and depending on the type of *CFTR* mutation, the risk for developing CP may increase 1.5- to 16-fold [75]. This risk increases 3- to 21-fold when *CFTR* mutations are combined with a mutation in *SPINK1*, which encodes the pancreatic secretory trypsin inhibitor [75]. The latter observation underlines the contention that pancreatitis is a multifactorial, complex disorder that develops through an interaction of multiple genetic and/or environmental factors. Consequently, the *CFTR* alleles that confer an increased risk of developing pancreatitis at the population level only have a small effect on the individual risk [76]. 

Typical environmental factors that, in conjunction with genetic factors, modify the risk of pancreatitis are alcohol consumption and inhalation of tobacco smoke. As many as 40 to 70% of CP patients drink excessive amounts of alcohol [77,78,79,80,81,82]. Similarly, smoking is an independent risk factor for the onset and recurrence of AP [83], and 60% of CP patients regularly consume tobacco [75]. *CFTR* mutations are more prevalent in alcohol- and smoking-related CP patients than in the general population, suggesting that partial loss of CFTR function aggravates the effect of such environmental factors [75]. Rosendahl et al. elegantly depicted the range of genetic and environmental interactions in different *CFTR*-associated diseases [63]. In CF and in hereditary forms of pancreatitis due to mutations in genes other than *CFTR* (such as *PRSS1*), genetics are dominant, and environmental factors have little influence on disease onset or severity. Conversely, for *CFTR* mutations that do not cause typical CF, the interplay with environmental risk factors becomes increasingly important.

To date, more than 2000 mutations in the *CFTR* gene have been described. These cover a wide spectrum, from apparently functionally silent mutations on one end, to the severe, CF-causing mutations on the opposite end of the scale. Information on the pathophysiological and clinical consequences of these variants has increased markedly over the last decade [1]. The diagnosis of CF requires two *CFTR* mutations on different alleles which severely impair CFTR channel function. Diagnosis of subjects who carry *CFTR* gene variants associated with residual protein function is often considerably less straightforward. The terms CF screen-positive, inconclusive diagnosis (CFSPID, European term) and CFTR-related metabolic syndrome (CRMS, North American term) describe patients with elevated immune-reactive trypsinogen who carry no or only one apparently CF-causing allele, which do not fulfil the diagnostic criteria for CF [84,85]. So-called CFTR-related disorders (CFTR-RD) are single-organ diseases with evidence of CFTR dysfunction in the absence of a CF diagnosis [1]. CFTR-RD are commonly associated with the presence of at least one *CFTR* allele with undefined clinical consequences.

### 3.1. Impaired Pancreatic Bicarbonate Transport in CF Patients

Impaired pancreatic HCO_3_^−^ secretion in CF patients was recognized far before the discovery of the disease-causing gene [86]. Follow-up studies indicated that both pancreatic HCO_3_^−^ and Cl^−^ secretion were strongly reduced in patients and contribute to the CF-typical fluid secretory defect [87,88].

As indicated previously, pancreatic dysfunction in CF is variable and correlates with genotype. In approximately 95% of the CF patients carrying severe *CFTR* mutations, i.e., resulting in an (truncated) immature protein that is not inserted into the plasma membrane (classes I, II), or in a channel with severely impaired gating (class III), the ion and fluid secretory function of the pancreatic duct is strongly impaired. This leads to atrophy of the ductal and, ultimately, also the acinar structures, culminating in extensive fibrosis and exocrine pancreatic insufficiency. In contrast, CF patients carrying “mild” class IV or V mutations are almost exclusively pancreatic sufficient [89]. Generally, pancreatic-sufficient CF patients also have a milder respiratory phenotype, lower mean sweat Cl^−^ concentrations and a higher life expectancy than pancreatic-insufficient CF patients [90]. The latter group of patients displays overt fibrosis of the exocrine pancreas and a virtually complete loss of HCO_3_^−^ and enzyme secretion. In the pancreatic-sufficient CF group, enzyme production is apparently adequate, but HCO_3_^−^ secretion may nevertheless be significantly diminished [91,92]. Pancreatic-sufficient CF patients are at an increased risk of developing pancreatitis, with a median age of onset of 18 years. Pancreatitis in this cohort is often precipitated by fatty meals and alcohol ingestion [93].

### 3.2. Bicarbonate and Viscid Mucus

It is well established that CFTR-dependent HCO_3_^−^ secretion is essential for solubilization of mucins, i.e., the polymeric glycoproteins that form the main constituent of mucus, in most CF-relevant epithelia [94,95]. In the respiratory and intestinal tracts, loss of CFTR-mediated HCO_3_^−^ secretion causes mucins to remain densely packed and attached to the epithelial surface. This leads to the formation of an abnormally viscid and strongly adherent mucus layer [96,97]. In the airways, reduced HCO_3_^−^ secretion and the concomitant accumulation of viscid mucus are also thought to affect pH regulation at the luminal surface, thought to be crucial for the defense against microbial colonization [98,99]. Furthermore, accumulation of hyper-viscid mucus impedes mucociliary clearance [100]. Thus far, the importance of HCO_3_^−^ in preventing mucus plugging and in anti-microbial defense in human pancreatic ducts has not been assessed, mainly because of the current lack of representative in vitro models and bona fide in vivo assays. However, ultrastructural and histochemical studies on autopsy pancreatic tissue and in newborn CF pigs and ferrets demonstrated that early acinar plugs consist of zymogen material, not mucus, but that subsequent mucous metaplasia occurs as the obstruction and exocrine atrophy progress [101,102]. Therefore, acinus plugging in CF is primarily due to a defect in ductal fluid secretion, not to accumulation of viscous mucus. 

### 3.3. Bicarbonate Transport in Subjects with Non-CF-Causing CFTR Mutations

CFTR-RD include diseases of the pancreas (i.e., acute recurrent or chronic pancreatitis), the male reproductive tract (congenital bilateral absence of the vas deferens) and the upper respiratory tract (i.e., chronic sinusitis) [103,104]. It has been suggested that, for proper function, these tissues are particularly dependent on CFTR-mediated HCO_3_^−^ transport, and that the non-CF-causing *CFTR* mutations associated with these disease syndromes primarily affect the capacity of CFTR to mediate HCO_3_^−^ transport [105,106]. Whether carrying a *CFTR* mutation on only one allele can give rise to these phenotypes is still unresolved [105].

In line with the concept that CFTR permeability can be dynamically regulated (Figure 2), it has been proposed that a select group of *CFTR* mutations specifically reduce the permeability of CFTR to HCO_3_^−^. Nine such mutations were identified (*CFTR* R74Q, R75Q, R117H, R170H, L967S, L997F, D1152H, S1235R and D1270N) in the North American Pancreatitis Study 2 cohort [105]. Patients were screened for *CFTR* mutations typically not associated with severe CF. These mutations were not only associated with pancreatitis but were additionally associated with rhinosinusitis and male infertility. They were termed bicarbonate-defective CFTR (CFTR^BD^) because they showed normal Cl^−^ but decreased HCO_3_^−^ permeability in response to WNK1/SPAK activation [105]. Two of these mutations, R74Q and R75Q, showed a reduced association with WNK1 [53]. However, more recent studies could not confirm that the R75Q mutation is a risk factor for CP, neither in the presence nor absence of a concurrent *SPINK1* mutation [107].

## 4. Acquired CFTR Dysfunction in Pancreatitis

Pancreatitis may not only be linked to *CFTR* mutations but may also result from environmental factors that reduce (wild-type) CFTR function.

### 4.1. Alcohol

Early studies showed that alcohol precipitates pain attacks in pancreatic-sufficient CF patients, as well as in patients suffering from hereditary pancreatitis and alcoholic CP [108]. Low concentrations of ethanol (0.3–30 mmol/L) acutely increased secretin-stimulated fluid secretion of guinea pig PDCs [109], while high concentrations (100 mmol/L) decreased ductal fluid and HCO_3_^−^ secretion by reducing CFTR expression and function [17,109]. Increased sweat Cl^−^ levels suggested that CFTR function was impaired in patients with excessive alcohol consumption [17]. CFTR protein expression was decreased in pancreatic tissues of AP and CP patients obtained from autopsies and surgical resections [17]. Ethanol or its metabolites (palmitoleic acid and palmitoleic acid ethyl ester) were also shown to decrease cellular cAMP and ATP levels in ductal cells, possibly by impairing oxidative phosphorylation [17,110,111]. Accordingly, inhibition of the carboxylester lipase, a key enzyme in the non-oxidative ethanol metabolic pathway, decreased the severity of alcoholic AP [112].

### 4.2. Bile Acids

Biliary pancreatitis is the leading cause of AP in both children and adults [113]. A proposed mechanism is the reflux of bile into the pancreatic duct, caused by gallstones or sludge within the distal common bile duct. Bile acid exposure causes pancreatic acinar cell injury through a sustained rise in cytosolic Ca^2+^ and activation of the Ca^2+^-activated phosphatase calcineurin [114]. Bile acids also exert a dose-dependent effect on PDC functions. A low concentration of chenodeoxycholate (CDC; 0.1 mmol/L) was shown to stimulate apical Cl^−^/HCO_3_^−^ exchange activity in guinea pig ducts [115] and CFPAC-1 cells only after *CFTR* expression [116]. However, CDC administration did not activate the CFTR Cl^−^ channel [115,116]. In contrast, a high CDC concentration (1 mmol/L) inhibited HCO_3_^−^ secretion in isolated guinea pig pancreatic ducts by causing severe mitochondrial damage [115,117].

### 4.3. Smoking

Smoking is associated with multiple systemic disorders and leads to acquired CFTR dysfunction in the airways, sweat glands and intestine [118]. The effects of smokers’ plasma on bronchial epithelial cells suggested the involvement of a circulating component of smoke [118]. Acrolein, nicotine, cadmium and manganese have been implicated in CFTR inhibition [118,119,120,121]. While some studies reported a transient activation of CFTR in airway epithelia [122], others showed rapid internalization of CFTR from the plasma membrane [123,124,125]. Importantly, the CFTR potentiator ivacaftor (VX-770) reversed the acute CFTR inhibition caused by cigarette smoke extract exposure in human bronchial epithelial cells [126]. 

Smoking is also an independent risk factor for the development of chronic pancreatitis [79]. Past and current smokers had lower secretin-stimulated peak HCO_3_^−^ concentrations in pancreatic fluid, indicating impaired CFTR-mediated pancreatic ductal secretion [127]. Cigarette smoke extract seemed to inhibit CFTR activity and HCO_3_^−^ secretion in guinea pig pancreatic ducts [119]. Smoking elevated sweat Cl^−^ concentrations in CP patients and decreased CFTR protein expression at the cell surface [128]. Incubation with cigarette smoke extract decreased CFTR expression in CAPAN1 cells and HCO_3_^−^ secretion in guinea pig pancreatic ducts [128]. Whether this decrease in HCO_3_^−^ secretion can be reversed by ivacaftor is not known but worthy of investigation.

### 4.4. Susceptibility to Pancreatitis Inducers

Since not all patients exposed to established environmental risk factors develop pancreatitis (e.g., the majority of heavy alcohol users do not develop pancreatitis), it is probable that additional (genetic) factors play a role. CFTR may be one of those genetic modifiers. Children diagnosed with pancreatitis who were exposed to smoke and had *CFTR* mutations were admitted to the hospital more often than children without *CFTR* mutations [129]. In patients with wild-type *CFTR*, a reserve CFTR activity may compensate for the deleterious effects of alcohol, but in patients carrying CFTR mutations, alcohol consumption may compromise CFTR function sufficiently to trigger symptomatic disease. Non-CF-causing *CFTR* mutations are also included in experimental pancreatitis models and underline the essential role of CFTR function in PDC (patho-)physiology. In transgenic mice with impaired Cl^−^ transport but significant residual CFTR function (CFTR^tm1HGU^), the severity of cerulean-induced pancreatitis was increased, mostly via impairment of PDC function and a shift towards a pro-inflammatory phenotype [130]. 

## 5. CFTR Modulators

Whereas symptomatic therapy of CF is only able to ameliorate the pathological consequences of CF, CF modulator therapy directly targets the roots of CF, namely, CFTR dysfunction [131,132,133]. Modulators are pharmacological compounds that can be classified on the basis of their different modes of action. So-called potentiators, e.g., ivacaftor (VX-770), restore CFTR channel gating; correctors, e.g., lumacaftor (VX-809), tezacaftor (VX-661) and elexacaftor (VX-445), improve CFTR folding and trafficking to the cell surface, and some such as elexacaftor may also act as co-potentiators [133]; amplifiers, e.g., PTI-CH, co-translationally enhance CFTR biosynthesis by stabilizing *CFTR* mRNA [134]; and read-through agents for *CFTR* nonsense mutations, e.g., aminoglycosides [135] and analogs (ELX-02) [136,137], or the recently developed compound SRI-37240, suppress premature termination codons [138]. The spectrum of mutations for which each modulator is clinically approved was reviewed recently [139].

### 5.1. Modulator Effects in the Pancreas

Thus far, only a few studies have addressed the effectiveness of CFTR modulators in animal models of pancreatitis. In a murine AP model induced by cerulean, pretreatment with tezacaftor and ivacaftor reduced the extent of tissue damage but did not affect other parameters, while in vitro administration increased fluid secretion in pancreatic ducts of AP animals [140]. Furthermore, in a murine autoimmune pancreatitis model, the CFTR corrector C18 rescued CFTR expression and localization in the pancreas [141]. Although these model studies provide valuable insight into the course of CFTR function and expression during pancreatitis, species differences should be considered, not only in epithelial physiology but also in drug sensitivity. For example, ivacaftor and multiple other potentiators have been shown to act on human but not murine F508del-CFTR [142].

Recent studies of ivacaftor with or without lumacaftor contradicted the assertion that exocrine pancreatic insufficiency is irreversible in CF children [143,144,145]. However, while pancreatic function improved, as evidenced by the increased fecal elastase-1 concentration and decreased immunoreactive trypsinogen levels, some studies reported an increase in pancreatitis episodes, resembling the phenotype of pancreatic-sufficient CF patients [143,144]. However, in pancreatic-sufficient CF adults, CFTR modulator therapy reduced the risk and frequency of RAP [146,147]. One study included 1800 CF patients who received CFTR modulators and showed a 67% and 62% reduction in AP hospitalizations in pancreatic-sufficient and pancreatic-insufficient CF patients, respectively [148]. These results suggest that early treatment with CFTR modulators might prevent pancreatic damage, and that CFTR modulators can ameliorate pancreatic complications of CFTR dysfunction. Interestingly, a recent case report documented a patient with RAP and an R117H/7T/F508del *CFTR* genotype without respiratory symptoms but with elevated sweat Cl^−^, who was effectively treated with ivacaftor [149]. This suggests a role for *CFTR* genotype testing in pancreatitis patients to identify those who could benefit from CFTR modulator therapy.

### 5.2. Modulator Effects on HCO_3_^−^ Transport

Multiple in vitro and a few in vivo studies have reported various effects of CFTR modulators on HCO_3_^−^ transport. For example, telemetric measurements of luminal pH in the duodenum of CF patients carrying the G551D gating mutation showed a profound increase in duodenal pH upon ivacaftor treatment, but in this study, the origin of HCO_3_^−^ (intestinal, biliary or pancreatic duct) was unclear [150]. In another in vivo study, ivacaftor/lumacaftor treatment of CF patients increased renal pendrin (*SLC26A4*)-mediated HCO_3_^−^ excretion through correction of F508del-CFTR activity in the β-intercalated cells of the collecting duct [37]. Interestingly, surface liquid alkalization in primary human airway epithelial cell cultures triggered by a triple combination of ivacaftor, elexacaftor and tezacaftor (marketed as Trikafta) was likewise pendrin mediated, but in this cell type, pendrin expression was dependent on pretreatment with the pro-inflammatory cytokines TNFα and IL-17 [151]. In Fischer rat thyroid cells, a common model to study CFTR function, the CFTR corrector lumacaftor was shown to increase the HCO_3_^−^ permeability of F508del-CFTR, and surprisingly to a greater extent than its Cl^−^ permeability [152]. Similarly, ivacaftor was reported to repair the specific HCO_3_^−^ conductance defect of the D1152H CFTR^BD^ mutation in monolayers of human nasal epithelial cells [153]. 

### 5.3. Modulator Studies in Pancreatic Ductal Organoids

**Figure 3:**
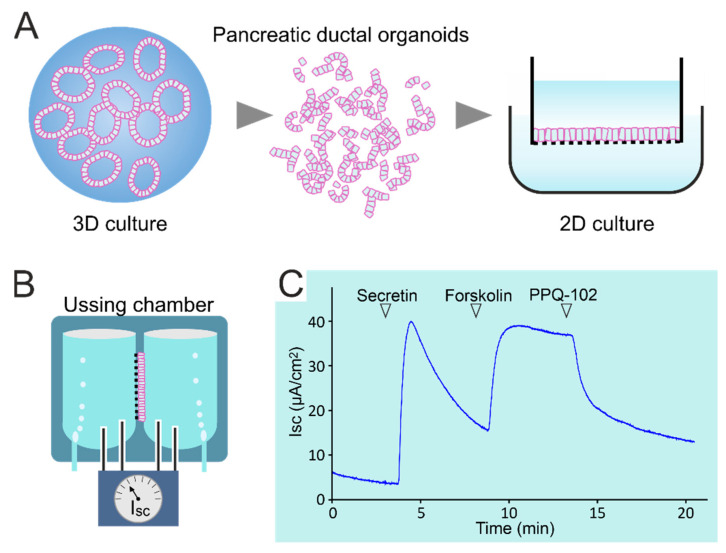
(**A**) Pancreatic ducts were isolated from a porcine pancreas by enzyme treatment and suspended in an extracellular matrix. From these ductal structures, spherical organoids are formed within a few days. Cultures are renewed and expanded by mechanical disruption and reseeding of the resulting cell clusters. These can also be used to initiate epithelial monolayers (2D cultures). (**B**) When cultured on a permeable substrate, monolayers can be used to assess epithelial anion transport in an Ussing chamber, analogous to ICM. (**C**) Current (Isc) response of a pancreatic organoid-derived monolayer upon stimulation by secretin and the cAMP agonist forskolin, and the effect of the CFTR blocker PPQ-102.

Organoids generated from stem cells in intestinal biopsies and cultured in an extracellular matrix (3D) or as monolayers (2D) on filters or on microfluidic chips have been used as novel tools to study epithelial (patho-)physiology and to predict clinical effects of CFTR modulators in individual patients (personalized medicine) [154,155,156]. Recently, culturing methods have been adapted to allow a similar long-term expansion of mouse and human pancreatic ductal organoids (PDOs) [157,158,159]. Pancreatic organoids can be generated from adult stem cells in pancreatic juice collected during endoscopic ultrasound [160], from microdissected animal- or patient-derived pancreatic ducts [159,161] or, possibly, even from microbiopsies collected during ERCP [162]. PDOs can be cryopreserved, clonally expanded and genetically manipulated and retain the characteristics of the tissue of origin, including gene expression and function of ion channels and transporters [157,161]. They can be grown 2D as polarized monolayers on filters or chips, offering highly suitable models to study Cl^−^ and HCO_3_^−^ transport pathways and their regulation [159,161] (Figure 3). However, it will be a challenge to reproduce the unique ability of PDCs to accumulate HCO_3_^−^ up to a 140 mmol/L concentration in luminal baths.

Human PDOs may also facilitate drug development and drug screening for pancreatic disorders such as CF or genetic and acquired forms of AP or CP. One restriction is the poor availability of human biopsies or tissue explants from the pancreatic duct of CF, CFSPID or CFTR^BD^ patients. In this case, creating a human model to study the effect of CFTR mutations and modulators on PDC properties and function would require gene editing of CFTR in non-CF PDOs. Additionally, it would be of great interest to study the ability of CFTR modulators to restore CFTR function in PDCs exposed to alcohol, tobacco smoke or bile salts. In particular, the therapeutic potential of VX-770 in a human PDO model deserves further investigation, considering the strong potentiating effect of this drug on wild-type CFTR channels [163].

## 6. Concluding Remarks

Whereas complete loss of CFTR function leads to the CF-typical fibrosis of the exocrine pancreas and pancreatic insufficiency, it has become clear in recent years that milder forms of CFTR dysfunction, whether congenital or acquired, are involved in the pathophysiology of pancreatitis. Congruently, recent studies suggest that CFTR modulators originally developed for CF therapy may also be of potential benefit in this context. However, not all patients suffering from pancreatitis carry CFTR mutations, and not all CFTR mutations may be amenable to correction. Therefore, it will be important to identify those CFTR variants that are potentially responsive to drug therapy. PDOs provide a promising model to achieve this objective, as they may be used to select modulators on a personalized basis. Finally, clinical testing of CFTR modulators is indicated to further clarify the role of CFTR dysfunction in the development of AP and CP, and to validate the therapeutic potential of CFTR modulators.

## Data Availability

Not applicable.
